# Biomechanics and Evolution of the Primate Tongue

**DOI:** 10.1002/evan.70026

**Published:** 2026-04-02

**Authors:** Yeganeh Sekhavati, Kaleb C. Sellers, Callum F. Ross

**Affiliations:** ^1^ Department of Organismal Biology and Anatomy University of Chicago Chicago Illinois USA; ^2^ Department of Oral Biology University of Illinois Chicago Chicago Illinois USA

## Abstract

Primate tongue morphology and function are critical to understanding the evolution of feeding, swallowing, and vocalization. In this paper, we examine the primate tongue as a muscular hydrostat with regionally specialized neuromuscular compartments. We integrate anatomical, kinematic, and biomechanical modeling approaches to analyze how muscle architecture and fiber orientation drive complex tongue deformations during functional behaviors. We evaluate the hydraulic mechanisms underlying tongue‐base retraction, highlight species‐specific adaptations in macaques and humans, and review primate tongue kinematics across distinct feeding stages. Finally, we synthesize recent advances in biomechanical modeling and experimental studies of tongue kinematics and their contributions to advancing three‐dimensional analyses of tongue movement during feeding and speech.

## 1 Overview

Biomechanical modeling of the primate tongue and oropharynx is crucial for testing hypotheses about structure‐function relationships during the evolution of chewing, swallowing, and vocalization [1]. Human swallowing biomechanics is also important for the treatment of dysphagia (disordered swallowing) and dysarthria (disordered speech) [2, 3]. Finite element analysis (FEA), MRI, and electromyography (EMG) are invaluable for quantifying tongue deformation during swallowing, chewing, and speech. These methods also provide critical insight into variation in primate hyoid arch morphology and neural control of tongue movements [4, 5]. This review synthesizes these anatomical, kinematic, and modeling approaches to understand how primate tongue structure underlies its diverse functional roles.

Despite decades of research, structure–function relationships in the primate tongue remain poorly understood. In this review, we argue that recent advances in imaging, modeling, and kinematic analysis make it possible to investigate these relationships. We propose a non‐reductionist view, suggesting that the primate tongue should be understood not as a collection of discrete muscles but as an integrated functional system. The primate tongue is an integrated muscular hydrostat and hydraulic system with regionally specialized neuromuscular compartments (NMCs) that coordinate to perform complex behaviors such as chewing, swallowing, and speech.

## 2 Primate Tongue Muscles Are Not Functional Units

**Figure 1:**
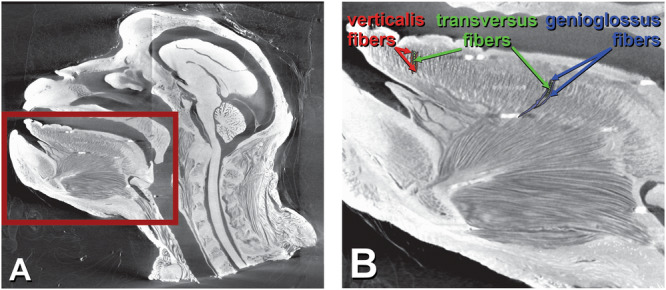
Parasagittal section of diffusible iodine‐based contrast enhanced micro computed tomography of *Macaca* illustrates how high‐resolution imaging reveals an interwoven muscle fiber architecture that challenges the traditional distinction between extrinsic and intrinsic muscle fibers. (A) Parasagittal section of head of *Macaca*. (B) Close‐up of tongue showing fibers of transversalis (green) coming in and out of the page interweave between fibers of verticalis (red) anteriorly and fibers of genioglossus (blue) posteriorly.

Tongue movement and shape change occur through muscle contractions and bulging, transmitted via connective tissue (CTs) and influenced by palate, jaw, teeth, hyoid and food. Vertebrate tongue muscles are grouped into intrinsic muscles, located entirely within the tongue (superior/inferior longitudinal [IL], transversalis, verticalis), and extrinsic muscles, which connect the tongue to external structures (palatoglossus [PG], styloglossus [SG], hyoglossus [HG], genioglossus [GG]) [1, 6]. However, separation into intrinsics and extrinsics does not accurately describe anatomy within the tongue. Many researchers have questioned the anatomical and functional validity of this distinction [7, 8, 9]. Histological studies show extensive overlap of intrinsic and extrinsic muscle fibers, such as verticalis, which includes fibers from extrinsic muscles such as GG and HG [10]. Our Dice CT data confirm that in *Macaca* GG fibers “replace” verticalis fibers posteriorly, interlacing between bundles of transversalis muscles (Figure 1). Within the tongue, muscle fibers act collectively and dynamically to alter tongue shape and position, regardless of whether they originate extrinsically or intrinsically. Rather than viewing the tongue as a collection of separate muscles, we endorse Schwenk's [10] view of the tongue as an integrated network of fibers that enables movements such as elongation, bending, and torsion. Recent work [7] supports this view, showing that lepidosaur tongues contain interwoven muscle fibers arranged orthogonally and circularly, functioning as an integrated unit. Rather than maintaining discrete anatomical boundaries, these fibers constantly shift between roles, such as lengthening the tongue during protrusion and bending to stabilize the bolus.

**Figure 2:**
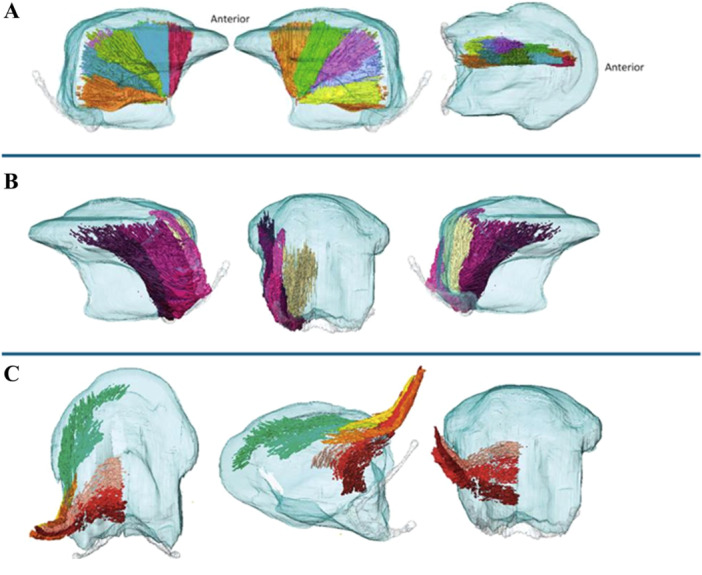
Neuromuscular compartments of the genioglossus, hyoglossus, and styloglossus muscles, based on Wrench [8]. NMCs are anatomically defined subdivisions within a muscle that represent a discrete subset of muscle fibers with distinct orientation, insertion and may function independently. (A) Genioglossus compartments illustrated in five sections: from left to right, sagittal view of the medial row, sagittal view of the lateral row, and axial view from above. (B) Hyoglossus shown in three compartments: from left to right, exterior sagittal view, posterior view, and interior sagittal view. (C) Styloglossus compartments illustrated in six sections: from left to right, axial view from above, view from below, and posterior view.

A recent study of human tongue anatomy also suggests that tongue function relies not on isolated muscles but on NMCs, subdivisions within muscles with distinct innervation, fiber types, and orientations [8] (Figure 2). Fiber orientations also cross traditional muscle boundaries: vertical and transverse fibers are common not only in the verticalis, transversalis muscles, but also in GG, while longitudinal, oblique, and helical patterns are found in superior/IL, SG, PG, and HG muscles [1]. Helical and crossed‐fiber patterns form the basis of the muscular hydrostatic skeleton. They maintain the tongue's shape while efficiently transmitting force. In this structure, fibers spiral around the tongue, enabling muscle contraction to produce complex motions [9]. This design allows the tongue to adjust force generation and transmission based on the task [9, 11, 12]. Interactions between muscle layers and helical fibers also allow elastic energy storage and release, likely improving efficiency during speech and feeding [9].

In simplified muscle models with fibers oriented predominantly along one axis, contraction involves fiber shortening accompanied by bulging in directions perpendicular to the fiber's axis [13]. However, this process becomes significantly more complex in structures like the tongue with fibers oriented along multiple axes [14]. These complex effects of muscle bulging during contraction are an essential component of the muscular hydrostat model of tongue function discussed below.

Most tongue muscles (except PG) are innervated by the hypoglossal nerve, whose motor nucleus is organized somatotopically. The rat hypoglossal nucleus is divided into a ventral subnucleus containing neurons innervating the geniohyoid and GG muscles, and a dorsal subnucleus housing neurons innervating the HG and SG [15].

Neural control of the tongue likely relies on coordinated muscle activation, often called muscle synergies [16]. Several kinematic pathways can lead to similar tongue shapes due to the tongue's biomechanical complexity. This variability supports the idea that synergies are not rigid patterns, but low‐dimensional motor commands linked to specific deformation modes. These “reusable deformation modules” likely originate in the brainstem, with afferent feedback modulating, but not fully determining, the resulting movements [17].

Proprioceptive afferents, including signals from muscle spindles, enter the central nervous system in the hypoglossal nerve (CN XII) but project centrally to the trigeminal sensory nuclei. Mechanosensory afferents from the anterior two‐thirds of the tongue are transmitted via the mandibular branch of the trigeminal nerve (V3), while those from the posterior third are carried by the glossopharyngeal nerve (CN IX). The facial nerve (CN VII) innervates taste buds in the anterior tongue, whereas the glossopharyngeal nerve (CN IX) serves the posterior tongue [18, 19, 20].

## 3 Anatomy and Function of Primate Tongue Muscles

Wrench [8] offers a key overview of human tongue muscles and their compartments. We briefly review these findings, incorporating additional data from Sanders et al. [21, 22, 23] and adult human tongue muscle volume from Stone et al. [24]. In our macaque specimens, we observe an organizational pattern consistent with Schwenk's [10] model of mammalian tongue structure, which features an outer cortex of longitudinally oriented fibers and an internal core of interlacing vertical and transverse muscles [25].

GG is the largest tongue muscle in humans. It originates from the superior mental spine of the mandible and fans out into the tongue body, with some of its lower fibers attached to the hyoid bone [1, 6, 26]. Humans may be distinguished from cats and other mammals by having GG fibers running into the tongue tip [10]. Our *Macaca* data set confirmed that although the GG extends anteriorly farther than in other nonhuman primates examined, its fibers do not reach the tongue tip. Structurally, GG is divided into multiple bundles to form thin laminae that interweave with the transversalis muscle laminae before penetrating the superior longitudinal (SL) layer to terminate in the lamina propria of the tongue's dorsum [1].

In humans, SG originates from the styloid process, runs antero‐inferior, passes superior and lateral to the HG before entering the posterolateral tongue [1, 6, 26]. There, it bifurcates: some fibers extending infero‐laterally, and others medially. SG likely contributes to tongue flexion and roll during chewing [27] and plays a key role in retraction and dorsum elevation in speech [28].

HG is a thin, quadrangular muscle originating from the greater horn and lateral hyoid bone, running forward and upward to enter the tongue, posterior to SG [1, 6, 29], where it comprises two nearly rectangular sections [26]. As HG approaches the SG border, its fibers form a thin sheet. This sheet then splits into smaller bundles that intertwine with SG fibers [1].

PG is the smallest extrinsic tongue muscle. It originates from the lower surface of the central tendon of the soft palate, descends antero‐inferiorly within the palatoglossal arch, and inserts into the tongue anterior to SG. From there, PG runs antero‐inferiorly to merge with SG [1, 6, 29]. PG is said to play a minimal role in tongue function [8], but it has important functions during suckling [30]; its morphology suggests bilateral contraction could raise the sides of the tongue, creating a trough for fluid transport.

The superior longitudinal muscle (SG) is a flat, superolateral sheet forming the tongue's uppermost layer, running antero‐posteriorly from the back to the tip [1, 6]. SL is a delicate membranous structure posteriorly that thickens toward the mid‐tongue before tapering again anteriorly toward its insertion [31]. Its fibers insert into the lamina propria at the tongue tip and terminate there, blending with fibers of SG and IL [25, 31]. The SL may be limited to the anterior two‐thirds of the tongue; in human fetuses, at the anterior edge of the tongue base (between the circumvallate papillae and the lingual tonsils) its fibers become sparse and give way to HG [25]. SL is thicker in the middle and medial portions of the tongue and gradually thins toward both the anterior and lateral margins [1]. When activated in concert with other lingual muscles, SL contributes to protrusion, retraction, and bending [32].

The IL muscle is a long, robust muscle originating at the tongue root and running anteriorly between GG medially and HG laterally in the posterior tongue, and between GG and SG in the anterior tongue. The IL muscle consists of two anatomically distinct compartments: a longitudinal compartment originating from the tongue root and inserting into the underside of the tongue tip; and an oblique compartment originating from the tongue root, inferior and medial to the longitudinal compartment, passing upward through the longitudinal part and inserting into the tongue dorsum near the SL muscle. Functionally, the longitudinal part enables tongue retraction and ventroflexion; the oblique compartment contributes to tongue bunching by controlling protrusion and preventing overextension [8].

The verticalis muscle (V) is a series of thin laminae originating from the ventrolateral submucosa, its fibers spreading out to pass through SL and attach to the lamina propria in the tongue's dorsum [1, 6]. Together with the T muscle, it is essential for shaping and elongating the tongue, primarily by compressing the longitudinal muscle fibers [33].

The transversalis muscle (T) originates from the median fibrous septum and extends laterally to insert into the supero‐lateral portion of the lamina propria. Confined to the central part of the tongue body, the muscle is arranged in serial laminae [1, 6]. It is typically described as the only transversely oriented tongue muscle [26], and it interdigitates extensively with the V muscle anteriorly and the GG muscle posteriorly [24]. However, in our *Macaca* specimens, we observed that some posterior GG fibers adopt an oblique trajectory, suggesting a partial transverse component. The T muscle fibers towards the tongue base are larger and have a higher density of spindles [34].

Across all tongue muscles, a few consistent organizational principles emerge. First, fibers are highly interdigitated and span traditional anatomical boundaries, undermining simple intrinsic versus extrinsic distinctions. For example, SG fibers merge with those of SL and IL muscles anteriorly, coursing together to the tongue tip, where the boundaries between SG, SL, and IL become indistinct [1]. Second, tongue muscles are organized into distinct NMCs (Figure 2), GG having the most (10) compartments. In contrast, muscles such as PG, SL, and IL have only two compartments each. These compartments differ in fiber type, innervation, and function. Electromyographic (EMG) studies show differences in activity across these compartments. For example, during swallowing, the anterior compartment of GG (GGa) activates earlier than the posterior compartment (GGp) [8]. Third, the interwoven architecture of longitudinal, vertical, transverse, and oblique fibers forms a mechanically robust system capable of supporting shape changes in multiple planes. We see this interwoven architecture, for example, in the V muscle, which runs dorsomedially to interweave with GG at the level of the T muscle. This collective organization supports the tongue's function as a muscular hydrostat. It also enables the hydraulic tongue‐base retraction (TBR) mechanism described in macaques.

## 4 Tongue CTs

The tongue's internal architecture is supported by a complex, well‐organized system of CTs that separates the muscle groups, allowing shearing between them [17], and providing anchorage for their fibers [35]. Furthermore, CTs transmit and resist forces for hydrostatic kinematics, so their anatomy, material properties, and function are of great importance, yet little is known about them. The lingual CTs include the tunica propria (a dense layer of CT just beneath the surface mucosa) and a submucous layer on the undersurface of the tongue [7, 31].

Several fibrous septa have been described in the human tongue. The median fibrous septum, which separates the left and right halves of the tongue, is most prominent in the anterior two‐thirds of the tongue but becomes thinner toward the tip and posterior root [31]. This septum provides attachment for the transverse muscles centrally, lies between the inner surfaces of the two GG muscles ventrally, and is almost absent dorsally, between the SL muscles [23, 31].

The paramedian septum is the most developed CT septum in the tongue, especially posteriorly. It is triangular, lying between GG medially, T and V muscles dorsally, and IL, HG, and SG muscles laterally [23, 31]. Its apex points anteriorly, while the posterior part becomes wider and attaches to the hyoid through the hyoglossal membrane. Medially, it connects to the median septum. The lateral surface of its posterior portion gives rise to the lateral septum. The paramedian septum also extends to the submucous layer and intermuscular septa on the floor of the mouth. It contains the lingual artery, accompanying veins, and a short segment of the hypoglossal nerve [31].

The lateral septum is located between ILM medially and HG and SG muscles laterally. It also is roughly triangular, with the apex facing anteriorly. The broader base is directed posteriorly and continuous with the posterior extension of the paramedian septum, which is part of the hyoglossal membrane. Its medial edge is attached to the paramedian septum. In its ventral portion, the lateral septum splits into medial and lateral lamellae that lie between ILM, HG, and SG. Inside the lateral septum is the lingual artery's main trunk and its dorsalis linguae branches. The glossopharyngeal nerve passes through the medial lamella, while the lateral lamella contains the hypoglossal nerve and its branches to SG, HG, and GG muscles. The lateral septum also contains accompanying veins (venae comitantes) and the lingual nerve [31].

In humans, the dorsal lamina propria gradually becomes thicker and stronger as it approaches the tongue tip. At the tip, it forms a cap‐like thickening, the anterior arch, which is more prominent ventrally than dorsally and which gives attachment to SLM, ILM, SG, HG, and GG. The lamina propria is also visible on the ventral side of the tongue, extending from the tip to about the point where the frenulum linguae attach [31].

## 5 Comparative Anatomy of Tongue and Suprahyoid Musculature

Variation in primate tongue musculature is poorly documented [1, 29]. Doran and Baggett [36] categorized mammal tongues into Type I (fleshy and spatulate and can only be protruded from the mouth up to 50% of its length), and Type II, (whip‐like, highly flexible, and protrusible). Type I is found in most mammals, including humans. Type II is found in myrmecophagous mammals. This categorization based on a single morphological axis of variation is unlikely to capture all of primate lingual variation.

Among hominids, the tongue musculature appears similar across taxa, although the full range of variation has yet to be described. The human tongue is rounder and is said to be more flexible than that of chimpanzees, enabling increased flexibility and unique shapes for the complex movements critical to human speech [1, 37, 38]. In contrast, the gorilla tongue retains the elongated, broad, and flat structure typical of macaques and gibbons, differing from the more oval chimpanzee tongue and rounded human tongue [37].

Chimpanzee tongue muscles are topologically similar to humans’. However, their flatter tongue is thought to emphasize protrusion and retrusion as primary actions, rather than the complex deformations typical of human tongues [1]. However, given the similarities between humans and macaques in tongue flexion and twisting during chewing [27, 39], and the apparent need for similar movements to control the food bolus during chewing in apes, tongue movements in non‐human hominids are certainly more complex than protraction and retraction.

Rhesus macaques (*Macaca mulatta*) are a well‐developed animal model for studying human swallowing physiology and neurobiology [5, 29]. In both macaques and humans, the tongue forms the floor of the oral cavity, extends through the fauces, and contributes to the anterior wall and floor of the oropharynx. However, in macaques, SG originates from the stylomandibular ligament, unlike in humans, where it arises from the styloid process, or in chimpanzees, where it originates from the petrous part of the temporal bone [1]. This difference gives the macaque SG a more horizontal orientation. In macaques, the stylohyoid originates from the styloid process and inserts into the inferior pole of the basihyal. In humans, it arises from the styloid process and inserts at the junction of the basihyal and the greater horn. The posterior belly of the digastric (D) originates from the mastoid process of the basicranium in macaques and from the mastoid notch in humans. The anterior belly attaches along the inferior border of the mandible for approximately half its length in macaques. In contrast, in humans, its attachment is restricted to the digastric fossa below the mandibular symphysis [40].

## 6 Hydraulic and Hydrostatic Models of the Tongue

Kier and Smith [11] proposed that the mammalian tongue functions as a muscular hydrostat: they are incompressible due to their high water content, they maintain a constant volume, requiring changes in one dimension to be offset by changes in others, and these shape changes can occur locally within the organ. In muscular hydrostats, fiber shortening generates movement by leveraging fluid incompressibility within the muscle fibers. When the tongue contracts in one dimension, it must expand in perpendicular dimensions to maintain a constant volume (reviewed by Ross et al. [5]).

In many ways, the tongue resembles a muscular hydrostat. It is a flexible organ composed entirely of muscles, lacking skeletal support, and maintaining a constant volume [11]. A key assumption of this classical model is that volume is conserved not only globally but also within specific regions. However, this assumption is challenged when applied to tongue base retraction (TBR), the backward movement of the tongue's posterior portion into the oropharynx, during swallowing. In humans, TBR is thought to result from combined shortening of extrinsic muscles (SG, HG) and intrinsic muscles (transversalis) [41]. In macaques, TBR involves an increase in volume of the posterior tongue and tongue base, driven by a hydraulic mechanism. All hydrostats incorporate hydraulics, but hydrostatics alone cannot explain why hydraulic forces cause one part of the tongue to increase in volume at the expense of others. The role of internal lingual anatomy must be considered.

Kinematic data suggest that in macaques, TBR is driven by elevation and forward movement of the hyoid bone into the oral cavity. This occurs through rotation and shortening of the mylohyoid and shortening of the geniohyoid [17]. The tissues in this space cannot accommodate the advancing hyoid. As a result, the midline tongue base and the food bolus are pushed backwards. This hydraulic mechanism relies on relatively small, slow hyoid movements that result in a larger and faster posterior tongue displacement. This amplification arises because the area of contact between the lower part of the tongue and the hyoid bone is broader than the area of contact between the posterior oral tongue and the tongue base. This difference in contact area creates a mechanical configuration in which small, slow hyoid movements produce larger, faster displacements of the posterior tongue [17, 29].

## 7 Experimental Studies on Tongue Kinematics

### 7.1 Electromagnetic Articulography (EMA)

EMA uses small transmitter coils, attached to the tongue surface, for real‐time tracking of their position and orientation. EMA offers high temporal resolution, especially useful for capturing rapid tongue movements during speech [42]. Its minimally invasive nature means that EMA can be applied to humans, and it has been widely used to analyze tongue biomechanics [43, 44]. EMA has been integrated with other imaging techniques. For example, EMA combined with MRI has enabled creation of dynamic 3D tongue models [45]. EMA data has also been used to estimate the motion of untracked tongue surface points by interpolating their positions from known sensor data, using both position and orientation information [46]. These studies demonstrate that EMA is uniquely suited to exploring the complexities of tongue movement.

### 7.2 X‐Ray Reconstruction of Moving Morphology (XROMM)

The development of XROMM significantly enhanced studies of tongue biomechanics by enabling precise three‐dimensional (3D) tracking of tongue, hyoid, and jaw movements during chewing and swallowing [47]. Historically, understanding mammalian tongue movement has been limited by the lack of high‐resolution 3D data [48]. In the XROMM workflow, movements of tantalum or zirconium beads implanted in tongue muscles are recorded from two perspectives using synchronized biplanar X‐ray imaging, allowing for detailed 3D reconstructions of tongue biomechanics [47]. The XROMM workflow has challenged traditional views on swallowing in primates, revealing a novel hydraulic mechanism [17, 29]. Feilich et al. [39] quantified 3D kinematics of tongue movements during chewing in macaques, showing that sagittal flexion and lateral roll are tightly synchronized with mandibular movements, particularly during the fast opening (FO) and fast closing (FC) phases of chewing. These coordinated actions, driven by intrinsic and extrinsic tongue muscles, are crucial for effective food manipulation and mastication.

Using XROMM, Laurence‐Chasen et al. [49] demonstrated that while overall tongue deformation patterns are largely unaffected by nerve block of oral tactile sensation, tongue‐jaw coordination variability increased. Narrowing of tongue width during jaw opening and prolonged fast‐opening phases highlights the critical importance of tactile feedback for efficient bolus manipulation and safe tongue repositioning during feeding.

Another way XROMM has transformed biomechanical research is by linking neural activity in the orofacial sensorimotor cortex with tongue movements [50]. This work reveals complex tongue deformations (e.g., rolling, flexion, protrusion) during feeding that are far more complex than those captured with 2D imaging techniques.

### 7.3 EMG

EMG is essential for studying lingual muscle activity during mastication, swallowing, and respiration [48], and is widely applied across a range of experimental contexts. Crompton et al. [51] used EMG with cinefluorography to track jaw and hyoid movements in opossums. It has been used to examine the contractile properties of intrinsic motor units in the rat tongue [52] and to clarify the roles of extrinsic and intrinsic tongue muscles. For example, it shows how GG controls protrusion, whereas the intrinsic muscles generate force [53]. Surface EMG also enables non‐invasive monitoring of supra‐hyoid activity, such as during tongue‐lifting exercises, designed to enhance control over tongue pressure without increasing the tongue's maximum force capacity [54].

EMG has been applied to swallowing studies, particularly to liquid foods. It can signal the elevation of the tongue, hyoid bone, and thyroid cartilage during swallowing [55]. It has also been used to study respiration. The GG muscle, for instance, is critical for maintaining airway patency, as evidenced by EMG recording [56].

However, interpreting EMG data without kinematic measurements can lead to misleading conclusions. EMG records the electrical activity of the muscles, indicating when a muscle is active, but it does not show whether the muscle is concentric (shortening), eccentric (lengthening), or isometric (static). For example, while the posterior mylohyoid is active during its shortening phase, it also exhibits low‐level activation toward the end of its lengthening phase, just before it begins to shorten [29]. Additionally, the anterior digastric muscle shortens passively, without being actively contracted. These findings highlight the importance of pairing EMG with XROMM and diceCT, which provide precise 3D measurements of muscle length and orientation.

## 8 Tongue Kinematics During Primate Feeding

We recently reviewed primate tongue kinematics during feeding [5], so our focus here is on areas where more research is needed. Hiiemae's Process Model of Feeding remains relevant for understanding feeding behaviors (biting, chewing, lapping), particularly due to its emphasis on tongue sensorimotor integration through feeding sequences [57, 58, 59, 60].

Feeding consists of cyclic jaw, tongue, and hyoid movements across stages: ingestion, stage I transport, processing, stage II transport, and swallowing. The gape cycle, defined by mandibular movements, is divided into fast close (FC), slow close (SC), slow open (SO), and fast open (FO) phases, which correspond to kinematic and sensorimotor transitions [60]. Detailed descriptions of these phases, their neural and kinematic correlates, and the roles of the tongue and hyoid are reviewed in Ross et al. [5]. In species with unfused mandibular symphyses, such as opossums, additional sub‐phases may be necessary to describe rotational movements [61]. Whether this applies to primates is unknown. As chewing progresses, changes in bolus properties are accompanied by alterations in jaw kinematics and muscle activity [62, 63].

### 8.1 Ingestion

Feeding begins with ingestion (bringing food or liquid into the mouth), which requires precise coordination of jaw and tongue movements. As the jaw opens, the tongue protracts to guide the bolus, then retracts during closure to aid transport while minimizing injury risk [64]. Some primates use specialized behaviors like gouging or hand‐assisted feeding [65]. Strategies for ingestion vary depending on the material: liquids are typically acquired through lapping, sucking, or licking, while solids require more pronounced protraction–retraction cycles and incorporate a SC phase [64] for bolus breakdown. Solid food ingestion also involves earlier tongue protraction and greater jaw yaw, producing longer, more rhythmic cycles. Tongue dorsiflexion is key to positioning solids for chewing [5].

Lapping, sucking, and licking each represent distinct strategies for liquid ingestion, adapted to different anatomical features and ecological needs. Lapping is characterized by rhythmic tongue movements that collect small volumes of liquid, a behavior commonly observed in animals with reduced cheek structures, such as cats and dogs [66, 67, 68]. In contrast, sucking is more common in species with well‐developed cheeks (like pigs and many primates) and depends on intraoral negative pressure to draw in fluid [69]. Licking, although mechanically similar to lapping, involves repetitive tongue contacts with the drinking source and is often used to sample moisture or other substances, effectively blending aspects of both lapping and feeding behaviors [70].

### 8.2 Stage 1 Transport

If food is deemed palatable, it is transported from the point of ingestion to the molars, Stage 1 transport [60, 66, 71]. In humans and macaques, this involves a “pull‐back” mechanism: during SO, the hyoid and tongue protract and lengthen, with the tongue tip cradling the bolus [27, 71]. As the jaw closes, the hyoid retracts, drawing the bolus posteriorly, aided by posterior tongue contraction in macaques [71, 72] and tongue base elongation in humans [58]. In macaques, which lack prominent palatal rugae, tongue protraction during Stage 1 transport is accompanied by twisting, likely to secure the bolus against the anterior cheek teeth [5, 60]. Differential contraction rates between tongue regions contribute to variations in transport velocity, suggesting regional control of tongue musculature [73].

### 8.3 Processing: Mastication and Tongue Palate Compression

Processing involves food breakdown via mastication or tongue–palate compression. As chewing progresses, tongue movements become increasingly precise, ensuring that the bolus remains on the occlusal surface for effective grinding and mixing with saliva [57]. The tongue performs dynamic lateral and twisting motions to reposition the bolus after each stroke, maintaining stability and cohesion [48, 57].

During this stage, tongue movements span both the sagittal (front–back) and coronal (side–side) planes. Protraction typically begins at the start of SC, with retraction occurring at FO. However, the timing varies with chew type. In simple chews, FO and tongue retraction begin at a minimum gape. In contrast, complex chews feature a prolonged SO phase, during which the tongue continues to protract until FO initiates retraction [5, 58]. In humans, the anterior tongue presses the bolus against the palate during SC and propels it posteriorly during FO.

Tongue twisting toward the chewing side helps guide the bolus to the post‐canine teeth, while cheeks stabilize the bolus laterally [74]. The tongue then rotates in the opposite direction to transfer food across the mouth [5, 39, 75].

### 8.4 Stage II Transport

Stage II transport refers to the movement of food or liquid through the fauces into the oropharynx, setting the stage for swallowing [58]. While most mammals use a “pull‐back” mechanism in which hyoid and tongue retraction during FO drive posterior transport, humans often rely on a “squeeze‐back” strategy: sequential tongue protraction beneath the bolus shifts tongue–palate contact rearward, advancing the bolus in a wave‐like fashion without moving the entire tongue backward [48, 57, 58, 66]. This mechanism is associated with anterior–superior hyoid motion during SO and FO phases [76]. Similarly, macaques also use the squeeze‐back mechanism, in which the bolus moves posteriorly into the oropharynx while the hyoid and tongue protract during the SO phase [72].

### 8.5 Bolus Formation and Deglutition

During later cycles in a feeding sequence on solid food, the tongue prepares the bolus for swallowing. When the bolus is assessed to be safe to swallow, starting around minimum gape and continuing through the SO phase, the hyoid elevates and protracts. The contact point between the tongue and palate slides posteriorly, gradually squeezing the food bolus into the oropharynx, which is a key feature of the hydraulic TBR mechanism [5, 17, 57]. TBR also flips the epiglottis and clears any residue [41].

During liquid swallowing, the tongue creates anterior and posterior seals to contain the bolus before initiating the swallow. Larger liquid volumes are accommodated by forming a deeper tongue cavity [77]. Swallowing begins with a coordinated, wave‐like tongue–palate press that propels the bolus posteriorly, while the tongue's back lowers to break the posterior seal, a process analogous to the squeeze‐back mechanism of solid food transport [57, 76]

Once in the pharynx, the pharyngeal stage begins: the larynx and velopharyngeal isthmus close, and the tongue base presses against the pharyngeal walls. Sequential pharyngeal contractions and elevation of the larynx and pharynx move the bolus caudally. Larger boluses are primarily propelled by tongue pressure; smaller ones by pharyngeal muscles [77]. Finally, hyoid protraction opens the esophageal entrance, and a pressure gradient created by the tongue and pharyngeal muscles moves the bolus into the esophagus [78].

For chewed solids, the bolus is not transported all at once as in liquid swallows. Instead, multiple Stage II transport cycles gradually move portions of food from the oral cavity to the oropharynx, a process known as oropharyngeal aggregation. Once swallowing is triggered, the tongue sweeps the remaining food into the pharynx (squeeze‐back), and the tongue base presses backward to propel the food through the pharynx (TBR) [58, 76].

## 9 Summary of Tongue Shapes in Feeding

### 9.1 Trough Shape During Ingestion and Stage I Transport

In humans and macaques, the tongue can create a bowl or trough‐like shape, with its center depressed and its sides elevated to hold and stabilize food during ingestion and Stage I transport. This shape differs from the pocket formation used for liquids, in which the tongue forms a small pocket at its tip. The trough‐like shape is hypothesized to be formed by GG and HG lowering the center of the tongue, while SG lifts its sides [27].

### 9.2 Twisting Shape for Bolus Placement and Stabilization

During mastication, the tongue flexes and twists sharply to one side, placing the food between the molars for food breakdown. Specifically, the middle part of the tongue twists toward the chewing side while the tip flexes toward the balancing side. These movements peak during the FC phase of chewing to align the food bolus with the teeth for mastication [27, 39].

Abd‐el‐Malek proposed that bending and twisting result from the contraction of the balancing‐side SG. However, analysis of the timing of these deformations suggests this is unlikely to be the only mechanism. In particular, the unilateral contraction of longitudinal muscles may also contribute to the flexion and twisting [39].

As the teeth come together to compress the bolus, the tongue maintains its twisted position, pressing its dorsum firmly against the medial side of the grinding teeth at the same time as buccinators press laterally, Abd‐el‐Malek's “guarding stage.” This prevents food from escaping into the oral cavity or vestibule. Intrinsic muscles, such as the transversalis and longitudinal fibers, may allow the tongue to adapt to the bolus shape. Meanwhile, SL adjusts the tongue tip or forms ridges to enhance stabilization [27]. It seems likely that there is precise feedback at this phase of the chewing cycle.

### 9.3 Sorting

As mastication progresses, the tongue manages and organizes food particles after each chewing cycle. The buccinator muscle pushes food from the cheeks toward the tongue. Larger, unchewed fragments are guided to the center of the tongue's trough for further grinding. Smaller, sufficiently processed pieces are moved to the side. This sorting and redistribution process continues until all food particles are broken down into sizes suitable for swallowing [27].

### 9.4 Lateral and Vertical Movements for Creating Cohesive Boluses

In the final stage of mastication, the tongue plays an important role in bolus formation, crushing food, mixing it with saliva, and coating it with mucus, creating a cohesive bolus. The tongue's precise movements, both lateral and vertical, ensure the bolus is uniformly prepared for swallowing [27]. These movements, documented by Matsuo and Palmer [79], are summarized below.

Vertical tongue movements (up and down) guide food onto the teeth for chewing, mix it with saliva, and shape it into a cohesive bolus ready for swallowing. The extent and timing of vertical tongue movements depend on food texture: harder foods require larger, more pronounced movements, while softer foods require smaller, more precise movements. To perform these movements effectively, the tongue must coordinate with the jaw. As the jaw depresses and elevates during chewing, the tongue moves downward to position the food and upward to stabilize or compress it against the palate or teeth. This coordination ensures efficient food breakdown and smooth swallowing.

Although the jaw drives these movements, the hyoid bone independently elevates the back of the tongue, especially during swallowing. Sorting also occurs during mastication as horizontal tongue movements help reposition the bolus within the mouth. The tongue moves the food forward or backward, ensuring fragments are aligned on the molars for efficient chewing and facilitating bolus formation.

Horizontal tongue movements are closely coordinated with jaw and hyoid motion, with the hyoid playing a more prominent role. While the temporomandibular joint restricts the jaw to primarily vertical and lateral movements, the hyoid moves more freely anteroposteriorly. This freedom allows the hyoid to assist the tongue by moving forward to squeeze the bolus toward the oropharynx during swallowing.

The tongue's anterior and posterior regions contribute differently to these movements. During the later stages of eating, particularly Stage II transport, the posterior tongue's anteroposterior motions, combined with the forward motion of the hyoid, create the pressure gradient needed to propel the bolus toward the pharynx efficiently. These movements result from the coordinated activity of intrinsic and extrinsic tongue muscles (particularly GG and HG). GG moves the tongue forward and HG pulls it backward. Meanwhile, intrinsic muscles such as IL and SL enable precise anterior‐posterior movements. The hyoid bone supports these actions by anchoring the extrinsic muscles and helping to pull the tongue back through its forward motion.

## 10 The Role of Tongue Muscles in Vowel Production

Feeding has clearly evolved before vocal communication. However, basal primates used the tongue for both behaviors. Many speech control strategies (such as precise shape modulation and region‐specific muscle activation) likely evolved from ancestral feeding mechanisms. At the same time, speech imposes unique demands, including rapid, sequential articulatory movements. The extent to which speech control is decoupled from feeding control is still unclear.

Speech production requires tightly coordinated actions by the respiratory system, larynx, and vocal tract (e.g., lips, tongue, and palate) [80]. Among these, the tongue changes in shape and position, particularly affecting the resonant frequencies, formants, crucial for distinguishing different vowel sounds [81].

Advances in MRI, tagged MRI, and FEA modeling have improved our understanding of tongue muscle function in speech articulation. These studies reveal that vowel production relies on both intrinsic and extrinsic tongue muscle activation and that articulation results from a coordinated interplay between multiple muscles [82]. Here, we limit our review to the roles of key tongue muscles in vowel production.

### 10.1 GG

GG is a primary driver of tongue motion during speech [32], with anterior (GGa) and posterior (GGp) portions contributing differently to tongue shaping for vowel production [82, 83]. GGp pulls the tongue root forward and elevates the dorsum, while GGa plays a key role in forming the central tongue groove [28]. These findings are consistent with EMG studies, which have identified distinct activation patterns of the GG during vowel production, including timing differences between front and back vowels [84].

Because different vowels require distinct tongue shapes and positions, the roles of these muscle regions vary depending on the specific vowel. GGp contributes to tongue advancement, whereas GGa shapes the anterior tongue surface, particularly for high‐front vowels like/i/(“ee” in seen). Localized activation of GGa plays a more significant role in shaping the tongue than the overall co‐contraction of GGp and GGa [28]. For high‐front vowels, GGp moves the tongue root forward, while GGa refines the groove. Additionally, tongue elevation and stabilization involve SG and MH. SG lifts the tongue, while MH stiffens the mouth floor to provide more stability during articulation [26, 28, 85].

For back vowels like/a/, GGa and HG work together to shape tongue movement. Rather than directly facilitating retraction, GGa limits apex rotation and increases the anterior cavity size. In contrast, HG pulls the tongue backwards and downwards while simultaneously rotating the tongue tip toward the palate. For/o/, a mid‐back rounded vowel, SG plays a key role by pulling the tongue backward and upward. Additionally, GGp expands the back cavity of the vocal tract by pushing the tongue forward and also contributes to the upward tongue movement essential for producing/u/.

For consonants, GGa contributes to tongue advancement in anteriorly produced fricatives like/s/, while GGp facilitates tongue retraction in posteriorly produced consonants like/k/ [82]. These findings confirm that GGp and GGa sometimes function “antagonistically,” with one region contracting while the other extends, allowing for precise motor control during articulation [86].

### 10.2 SG

SG plays a key role in tongue retraction and dorsum elevation, particularly during the articulation of high‐back vowels like/u/. SG pulls the tongue tip downward and backward while elevating the dorsal tongue. This movement, in coordination with GGp, helps regulate the constriction location necessary for producing high vowels like/u/ [26, 28, 86, 87].

### 10.3 HG

HG is responsible for tongue depression, particularly during the articulation of low‐back vowels like/a/ [28, 83, 85]. When activated, it pulls the tongue downward and backward, sometimes causing the tongue tip to rotate toward the palate. This movement influences the size of both the anterior and posterior vocal tract cavities. EMG consistently shows that HG activation occurs during low vowel articulation, reinforcing its role in controlling tongue position [87].

Although HG operates within a coordinated muscular system, especially when working with GG, its activation patterns can vary across individuals [82]. While it frequently contracts during the articulation of low vowels like/a/and sometimes functions alongside GGp, its role appears less consistent than that of the GG or transversalis (T) muscles [88].

### 10.4 SL and IL Muscles

SL and IL muscles can play complementary or opposing roles in tongue movement. IL lowers and retracts the tongue tip, while SL elevates and retracts it. Additionally, SL is closely linked to GGa and HG, suggesting its role in overall tongue positioning. These muscles are particularly important for dental and alveolar plosives like/t/and/d/, with SL elevating the tip and IL counterbalancing it [28, 82].

Although both muscles contribute to tongue retraction, IL plays a more prominent role in sounds like/u/and/k/. While IL activation patterns are consistent across speakers, SL activity varies, especially during phoneme transitions [82]. Further evidence of SL's role in articulation comes from simulations showing that its activation leads to tongue tip curling and slight retraction, contributing to alveolar consonants such as/t/. However, SL alone is not sufficient for full articulation; precise tongue shaping requires coordination with other muscles, such as the verticalis and transversalis [26].

### 10.5 Transversalis (T) and Vertical (V) Muscles

T and V contribute differently to vowel production. T maintains the tongue's transverse dimension and influences its midsagittal shape during speech. Earlier speech models [89] overlooked the role of T, assuming that the tongue could not compress in the sagittal plane. However, more advanced 3D modeling has since demonstrated that T plays an indirect but significant role in midsagittal tongue shaping, particularly by preventing excessive widening during high anterior vowels like/i/ [28].

V on the other hand, contributes to tongue flattening and widening. Simulations have shown that its contraction reduces tongue height while increasing lateral width [85]. While its direct impact on vowel shaping appears limited, V may assist in fine‐tuning tongue posture and contribute to overall stiffness, thereby influencing the actions of other muscles during articulation [28].

T and V show opposing strain patterns during speech, as evidenced by their negative correlation in fiber‐aligned strain waveforms [82]. Specifically, T, V, and possibly GG work together to facilitate anterior tongue protrusion. V flattens and widens the tongue, enabling elongation, while T narrows and extends it. GG may also contribute to this process, but its role in humans appears to be less significant compared to other mammals, where strong tongue extension is essential for feeding [14, 32].

## 11 FEA Analyses of Tongue Biomechanics

FEA is becoming increasingly important in studies of tongue biomechanics. It is an effective tool for simulating complex muscle interactions and deformations associated with speech, swallowing, and other orofacial functions [32, 88]. By modeling muscle fibers and their orientations, FEA can illustrate the effect of muscle activation on tongue shape and position [90].

FEA is particularly valuable for analyzing tongue muscle coordination in precise movements. Stavness et al. [32] used FEA to predict muscle activation patterns during protrusion and bending. Their research demonstrated that anterior protrusion involves bilateral activation of GG, T, and V. In contrast, SL counteracts downward forces, emphasizing the importance of coordinated muscle contractions for shaping and controlling tongue movements [32, 82].

FEA can also be applied to the study of the tongue during speech. Sanguineti et al. [91] created a validated biomechanical model of the human tongue, emphasizing muscle‐level controls involved in speech movements. They demonstrated that the complex tongue motions during speech could be accurately simulated and managed using FEA. Their study also supported the hypothesis that the central nervous system employs simplified control strategies, relying on a limited set of independent muscle synergies to govern tongue movements.

Several studies have examined the dynamic interactions between the tongue and surrounding structures, such as the jaw, teeth, and palate. Hannam et al. [92] incorporated these interactions into a model to simulate chewing dynamics and load‐bearing activities. Their dynamic simulations, which included jaw and hyoid biomechanics, aligned with experimental observations [48, 93].

FEA can also play a crucial role in clinical settings. For example, it has been used to simulate tongue movements pre‐ and post‐glossectomy [85]. By simulating how surgical interventions may affect tongue mobility and control, which are critical for patients' quality of life after surgery, the model helps predict functional outcomes. FEA can also be used to investigate pathological conditions that affect tongue function, such as obstructive sleep apnea and muscular atrophy. Amatoury et al. [94] used 2D FEA to model airway dynamics in rabbits, demonstrating how tongue positioning and muscle tone contribute to airway obstruction. These models can be adapted to simulate similar conditions in humans and develop therapies for sleep‐disordered breathing.

Future directions in FEA research could address several critical yet unanswered questions about the tongue's muscular coordination, control, and response to pathological conditions. An important future research area will be to understand the precise, dynamic interaction between intrinsic and extrinsic muscles during complex movements like speech and swallowing. Although FEAs can isolate individual muscle functions, the coordination of multiple muscle groups under varying loads remains poorly understood.

## 12 Conclusion

In this review we examined the anatomy, function, and biomechanics of the human tongue, focusing on its intrinsic and extrinsic muscles and their roles in chewing, swallowing, and speech. The complex interactions between these muscles were further explored, emphasizing how compartmentalization enables highly precise movements. We evaluated hydraulic and hydrostatic models as key frameworks for understanding tongue function and discussed how methods such as FEA, EMA, and XROMM will continue to contribute to tongue neuromechanics research. Future research on the evolution of primate tongues would benefit from detailed anatomical and in vivo studies during feeding and vocalization to better understand trade‐offs between these functions.

## Conflicts of Interest

The authors declare no conflicts of interest.

## Data Availability

The authors have nothing to report.
